# NVP-LDE225, a Potent and Selective SMOOTHENED Antagonist Reduces Melanoma Growth *In Vitro* and *In Vivo*

**DOI:** 10.1371/journal.pone.0069064

**Published:** 2013-07-30

**Authors:** Ahmad Jalili, Kirsten D. Mertz, Julia Romanov, Christine Wagner, Frank Kalthoff, Anton Stuetz, Gaurav Pathria, Melanie Gschaider, Georg Stingl, Stephan N. Wagner

**Affiliations:** 1 Division of Immunology, Allergy and Infectious Diseases, Department of Dermatology, Medical University of Vienna, Vienna, Austria; 2 Novartis Institutes for Biomedical Research, Vienna, Austria; 3 Center for Molecular Medicine of the Austrian Academy of Sciences, Vienna, Austria; University of Navarra, Spain

## Abstract

Melanoma is one of the most aggressive cancers and its incidence is increasing worldwide. So far there are no curable therapies especially after metastasis. Due to frequent mutations in members of the mitogen-activated protein kinase (MAPK) signaling pathway, this pathway is constitutively active in melanoma. It has been shown that the SONIC HEDGEHOG (SHH)-GLI and MAPK signaling pathway regulate cell growth in many tumors including melanoma and interact with each other in the regulation of cell proliferation and survival.

Here we show that the SHH-GLI pathway is active in human melanoma cell lines as they express downstream target of this pathway *GLI1*. Expression of *GLI1* was significantly higher in human primary melanoma tissues harboring BRAF^V600E^ mutation than those with wild type BRAF. Pharmacologic inhibition of BRAF^V600E^ in human melanoma cell lines resulted in decreased expression of GLI1 thus demonstrating interaction of SHH-GLI and MAPK pathways. Inhibition of SHH-GLI pathway by the novel small molecule inhibitor of smoothened NVP-LDE225 was followed by inhibition of cell growth and induction of apoptosis in human melanoma cell lines, interestingly with both BRAF^V600E^ and BRAF^Wild Type^ status. NVP-LDE225 was potent in reducing cell proliferation and inducing tumor growth arrest *in vitro* and *in vivo*, respectively and these effects were superior to the natural compound cyclopamine.

Finally, we conclude that inhibition of SHH-GLI signaling pathway in human melanoma by the specific smoothened inhibitor NVP-LDE225 could have potential therapeutic application in human melanoma even in the absence of BRAF^V600E^ mutation and warrants further investigations.

## Introduction

Human melanoma is the most malignant skin cancer [1]. The incidence of malignant melanoma is increasing in general population and is the most common skin cancer in females aged 25–29 [2]. So far there are no effective therapies after metastasis. Human melanoma is a heterogeneous cancer. Depending on body site and extent of sun exposure it is characterized by different genetic alterations [3]. Frequent mutations in signaling pathways responsible for growth and proliferation of melanoma have been discovered [4]. Among these pathways the MAPK-cascade seems to be the most important one [5]. Activating mutations in two of MAPK upstream regulators, BRAF and NRAS is seen in up to 80% of melanomas and also correlates with melanoma aggressiveness and poor prognosis [6], [7], [8]. These molecules or their downstream kinases (among others MEK 1/2) are potential therapeutic targets for human melanoma. Recent discoveries in this field has resulted in the development of multiple drugs, among them vemurafenib (PLX-4032) a small molecule inhibitor targeting mutated BRAF^V600E^ (a mutation seen in 60% of human melanomas). A pilot clinical study by Fleherty K. et al. [9] showed an 81% response rate in stage IV melanoma patients refractory to conventional chemo/immunotherapies. Interestingly, in a recent phase 3 randomized trial comparing vemurafenib with dacarbazine, vemurafenib yielded improved rates of overall and progression-free survival in patients with previously untreated melanoma with the BRAF^V600E^ mutation and was superior to dacarbazine [10]. Several clinical trials are testing the effectiveness of MEK 1/2 inhibitors in melanoma patients (www.clinicaltrials.gov/ct2/results?term=MEKmelanoma). A pilot study presented by S. P. Patel at the 2010 ASCO meeting in Chicago showed a trend toward response and clinical benefit in the patients treated with MEK 1/2 inhibitor and harboring a BRAF^V600E^ gene mutation [11]. High incidence of relapse in these patients after an initial phase of clinical response argues for development of possible new mutations or skewing of the BRAF-MEK-ERK signaling pathway into a BRAF-independent NRAS-PI3K-AKT-mTOR or c-RAF-MEK-ERK pathways [12], [13], [14], [15]. Therefore, identification of new targets for possible combination therapy is inevitable.

HEDGEHOG (Hh) is a polypeptide, intercellular signaling molecule. It was initially discovered in drosophila and is known to play crucial role as segment polarity gene [16]. Hedgehog signaling also plays an important role in fly embryogenesis [17] and other developmental processes [18]. Three hedgehog homologues have been reported in mammals: SONIC-Hh, INDIAN-Hh and DESERT-Hh, with SONIC-Hh being most extensively studied [19]. Although Hh signaling is imperative to the normal human development and other vital processes [20], aberrant Hh signaling has been shown to cause developmental defects and promote tumorigenesis [21], [22].

PATCHED, a 12 transmembrane protein is the receptor for Hh, which in its active, unbound state binds and inhibits SMOOTHENED (Smo), another seven-transmembrane protein. SMOOTHENED is a positive regulator for the activation of GLIoma-associated (GLI) family of Zinc finger transcription factors activation and their subsequent nuclear translocation [23], [24]. GLI transcription factor family comprises of three members: *GLI1*, GLI2 and GLI3. GLI1 and GLI2 are transcription activators whereas GLI3 has been suggested to function as a transcription repressor [25]. *GLI, PTCH1, NOTCH*, *WNT*, platelet-derived growth factor, insulin like growth factor, fibroblast growth factor have been shown as some of the Hh target genes, regulated by transcriptional activity of GLI [26], [27], [28], [29], [30].

Activating mutations in the components of Hedgehog signaling or elevated Hh levels enhance the signaling output of this pathway in several cancers. Mutations in different signaling molecules within Hh-signaling pathway have been identified in sporadic medulloblastoma [31], [32], while inactivating *PTCH1* mutations have been associated with Gorlin-Goltz Syndrome [33]. Patients suffering from Gorlin-Goltz Syndrome develop basal cell carcinomas and carry much higher risk of developing medulloblastoma and rhabdomyosarcoma. Inactivating *PTCH1* mutations have been attributed to most of the sporadic BCC whereas *SMO* mutations account for approximately 10% of the cases [34], [35]. Although mutations in the Hh signaling pathway could account for pathology of some of the cancers, there has been constant increase in the belief that enhanced Hh levels in the tumor-microenvironment could also play a pathogenetic role in promoting several other types of cancers. Elevated Hh levels and enhanced expression of Hh target genes has been detected in diverse cancer types, such as pancreatic cancer, small cell lung cancer, gastric cancer, upper gastrointestinal cancer, pancreatic cancer and prostate cancer [22].

Until recently the involvement of Hh signaling in melanomas was unknown and unexpected due to the lack of genetic perturbations or enhanced expression of the Hh signaling components [36]. Recently the hedgehog signaling requirement has been shown in melanoma cell lines and in genetically induced melanoma mouse model [37]. In this study, authors show that hyperactivated Mek-Erk and Akt signaling could enhance transcriptional activity of *GLI* [37]. The SHH-GLI and MAPK signaling pathways also regulate growth in many tumors, suggesting cooperation between these two pathways in the regulation of cell proliferation and survival [38], [39], [40].

In line with the importance of hedgehog signaling in tumor biology, a range of drugs have been developed in the last years to block this signaling pathway. Cyclopamine and Jervine, derived from corn lilies are among the initial compounds discovered with potent inhibitory activity against SMOOTHENED [41], [42]. High-throughput screening of small molecule libraries has led to the identification of several additional SMO inhibitors including HhAntag [43], SANTs1-4 [44], Cur-61414 [45] and NVP-LDE225 [46].

In this study, using quantitative real-time PCR and cDNA microarray analysis, we show that the *GLI1* is expressed in human melanoma cell lines and its expression is significantly higher in primary human melanoma tissues harboring BRAF^V600E^ mutation as compared to those with wild type BRAF. Inhibition of BRAF^V600E^ using specific inhibitor PLX-4032 resulted in significant reduction in the expression of both GLI1 and phospho-ERK 1/2 at protein level. We demonstrate that both standard SHH-GLI inhibitor cyclopamine and the novel more specific inhibitor of smoothened NVP-LDE225 reduce the *GLI1* promoter activity, induce G1 cell cycle arrest, and induce apoptosis in human melanoma cell lines. Finally, the *in vivo* antitumor activity of NVP-LDE225 in human melanoma xenotransplantation model was potent and significantly higher than cyclopamine.

## Materials and Methods

### Mice

6–10 weeks old athymic Nude-Foxn1 nu/nu mice (Harlan Winkelmann, Borchen, Germany) were used in the experiments. All experiments were done with approval and following the guidelines of the Animal Research Committee of the Medical University of Vienna and the Austrian Ministry of Science and Research.

### Cell lines, tissues and reagents

Normal Human Epidermal Melanocytes (NHEM) were obtained from Promo-Cell (Heidelberg, Germany) and cultured in Melanocyte Growth Medium M2 (Promo-Cell). Human melanoma cell line MALME 3M, SK-MEL-2, SK-MEL-3, SK-MEL-5, SK-MEL-28, HT-144 and MEWO were obtained from American Type Culture Collection (Manassas, VA). UACC-62, 257, M14 cell lines were from DCTD Tumor Repository (National Cancer Institute, Frederick, MD). WM 35, WM 115, WM 165-1, WM 266-2, WM 278, WM 983A, WM 983B, WM 983C cell lines were kindly provided by Dr. Meenhard Herlyn (Wistar Institute, Philadelphia, PA). These cell lines have been published before and characterized by genomic and immunology approaches [47], [48]. MEL FH was a gift from Professor Nick Hayward (Queensland Institute of Medical Research, Australia) [7], [49]. On receipt, the authenticity of cell lines was verified using cytology and flow cytometry, throughout the culture by assessment of typical morphology by the investigators and, whenever indicated, by sequencing for the presence of gene mutations. Mutational status in several relevant oncogenes or tumor suppressor genes is demonstrated in Table S1.

Cells were cultured in RPMI-1640, supplemented with 2.5% heat-inactivated FCS (Fetal Calf Serum) and 2 mM L-glutamine (all from Invitrogen, Carlsbad, CA) and hereafter referred to as culture medium. After seeding from cryopreserved stock, cells were passaged at least 2–3 times before experiments.

NVP-LDE225 was provided by Novartis Austria and cyclopamine was from Merck KGaA (Darmstadt, Germany).

Propidium iodide (PI, Sigma-Aldrich, Vienna, Austria), Annexin V (Applied Biosystems, Foster City, CA), DMSO (Dimethyl sulfoxide) and thymidine (both from Sigma-Aldrich) were used in this study.

### RNA isolation, gene expression profiling and quantitative real-time PCR (RT-PCR) analysis

Total RNA was isolated from monolayer cell cultures and cryopreserved tissues using TriReagent (Sigma-Aldrich) according to the manufacturer's instructions.

Tissue sampling and gene expression profiling was done by using Affeymetrix U133A microarray platform as previously described [50]. Microarray experiments were conducted according to standard protocols for Affymetrix Genome U133A arrays (Affymetrix, Inc., Santa Clara, CA). Briefly, 1 µg of total RNA was used to synthesize cDNA and biotinylated cRNA using the GeneChip expression 3′ amplification reagent kits of Affymetrix. cRNA hybridization and scanning of the array were performed according to the manufacturer's protocols. Gene set and pathway enrichment analysis has been performed as previously described [50]. The data have been deposited in the National Center for Biotechnology Information GEO [51] and are accessible through GEO Series accession no. GSE8401.

For RT-PCR study reverse transcription was done by SuperScript II reverse transcriptase using random hexamer and oligo-dT primers (all from Invitrogen). GLI1, β-actin cDNAs or 18S rRNA were amplified using primer/probe sets # Hs01110770_g1, # Hs99999903-m1 and # Hs99999901-s1, respectively from Applied Biosystems. Reactions were performed in an AbiPrism 7700 DNA amplifier under following conditions: initial heating at 50°C for 2 min and 95°C for 10 min, followed by 40 cycles consisting of a denaturation step (90°C, 15 s) and an annealing/elongation step (60°C, 1 min). Each sample was assayed in duplicate. Negative controls represented full PCR reaction mixtures with cDNA substituted by ddH_2_O. The read-out for each reaction was cycle of threshold (Ct), i.e. the cycle number where the fluorescent signal became higher than a pre-defined threshold.

In all experiments, levels of *GLI1* mRNA were expressed semi-quantitatively using the ΔΔCt method and 18S rRNA as a reference gene, according to the formula:
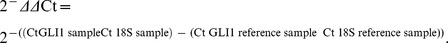



### pGL3b-hPTCH1-prom-wt and pGL3b-hPTCH1-prom-mut Promoter Reporter Construct

Specially designed reporter constructs were kindly provided by Dr. Fritz Aberger (Department of Molecular Biology, University of Salzburg, AT). Plasmids were constructed on the basis of pGL3-basic vectors (Promega, Madison, WI) with a firefly luciferase reporter gene. The wild type PTCH1 (PTCH1-wt) reporter plasmid was constructed with the human wild type *PTCH1* promoter with two functional GLI binding sites (5′-GACCTCCCA-3′ and 5′-GACCACCCA-3′, see Figure S1). This was shown to be a consensus target sequence for all three GLI transcription factors (TFs) [52]. A control reporter construct (*PTCH*-mut) was generated by introduction of a CC>GG substitution (GACCTCCCA>GACCTGGCA and GACCACCCA>GACCAGGCA). This mutation prevented binding of GLI TFs to the promoter sequence (personal correspondence with Dr. Fritz Aberger, 2009/04/11). Subclones were verified by DNA sequencing (VBC-Genomics, Vienna, Austria). Transfection efficacy was determined by co-transfection of a pRL-TK promoter reporter construct (Promega). The plasmid contains the Renilla luciferase reporter gene under the control of a HSV-TK (herpes simplex virus-thymidine kinase) promoter, which provides constitutive expression in mammalian cells [53], [54].

### Dual Luciferase Reporter Gene Assay

Luciferase activity was measured after transient transfection of the pGL3-basic vector (determination of background luciferase activity), the pGL3b-hPTCH1-prom-wt and pGL3b-h*PTCH1*-prom-mut. Transfection efficiency was normalized by cotransfection with a pB-actin-RL reporter containing a full-length renilla luciferase gene under the control of a human β-actin promoter. For transfection experiments, 2×10^5^ cells were cultured overnight in 48 well tissue culture plates. Luciferase reporter plasmids were added together with Lipofectamine™ 2000 according to manufacturer's protocol (Invitrogen). All experiments were performed in triplicates. Measurement of luciferase expression was done according to manufacturer's protocol (Promega).

### Immunofluorescent microscopy

The immunofluorescent microscopy was performed as previously described [47].

### Cell cycle analysis, Annexin V and MTT assays

Cell cycle analysis was performed as elsewhere described [55]. Briefly, on specific time points cells were collected, resuspended in PBS (Phosphate Buffered Saline, Invitrogen), ice-cold 70% ethanol (Sigma-Aldrich) added dropwise while mildly vortexing. After removing ethanol, cells were washed with 0.05% Tween-20 (Sigma-Aldrich) in PBS and then resuspended in DNA staining buffer [(PBS/PI 10 µg/ml/RNase A 250 µg/ml (Sigma-Aldrich)]. After 15 min incubation at 37°C PI signal was acquired in FL-2 or FL-3 channel of a flow cytometer using a linear scale (FACScan, BD Biosciences, Vienna, Austria). Hypodiploid (necrotic/apoptotic), diploid (G1/G0) and tetraploid (G2/M) cells were quantified using CellQuest software (BD Biosciences).

For annexin V-based apoptosis detection assay, human melanoma cell lines were collected at desired time points post drug treatment. After washing in PBS (Invitrogen) cells were resuspended in 1×Binding buffer (BD Biosciences), annexin V-FITC (BD Biosciences) and PI were added according to the manufacturer's protocol (BD Biosciences). After 15 min incubation at room temperature, cells were acquired by flow cytometry. All, early apoptotic cells (annexin V positive, PI negative) and necrotic/late apoptotic cells (annexin V positive, PI positive) as well as living cells (double negative) were detected by FACScan and subsequently analyzed by CellQuest software (BD Biosciences).

Standard MTT (3-(4,5-Dimethylthiazol-2-yl)-2,5-diphenyltetrazolium bromide) assay was performed in 96-well plates as described previously [56]. The viability was measured using following formula:
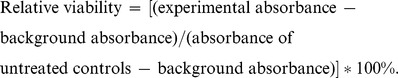



### Western blot analysis

Antibodies were rabbit anti-human GLI1 (100-401-223, Rockland Immunochemicals, Gilbertsville, PA), Phospho-p44/42 Erk1/2 (Thr202/Tyr204, E10) (Cell Signaling Technology, Danvers, MA), and α-tubulin (DM1A) (Calbiochem, Darmstadt, Germany). For western blotting, equal amounts of protein, as determined by the BCA protein micro assay (BioRAD, Hercules, CA), were resolved by sodium-dodecyl sulfate-polyacrylamide gel electrophoresis (SDS-PAGE). Primary antibody staining was done at 4°C over night. Visualization was done using the ECL Detection System (Pierce, Rockford, IL).

### Tumor xenografts

1×10^6^ tumor cells were inoculated subcutaneously into 6- to 10-week-old nu/nu athymic female mice into both flanks in 100 µl of PBS containing 10% FCS. Tumors with the mean volume of 48+/−4 mm^3^ were injected with vehicle (PEG 300/10% EtOH), cyclopamine or NVP-LDE225 on daily basis.

Tumour growth was monitored every 2–3 days and the volume calculated (π/6×(larger diameter×(smaller diameter)^2^). Mice were observed daily for survival and body weight measured. Experiments were terminated when tumors reached volumes >1500 mm3 or animals showed signs of distress due to tumor growth. All experiments were done with approval and following the guidelines of the Animal Research Committee of the Medical University of Vienna and the Austrian Ministry of Science and Research.

### Statistics

Statistical analysis was performed using GraphPad-Instat™ software (www.graphpad.com). *GLI1* expression in tissues was analyzed by ANOVA test with Tukey's post-test. Growth of human tumor xenografts and human primary melanoma transcript levels were analyzed using two-sided Welch's t-test.

## Results

### *GLI1* expression is significantly higher in human primary melanomas harboring BRAF^V600E^ mutation as compared to BRAF^Wild Type^

**Figure 1:**
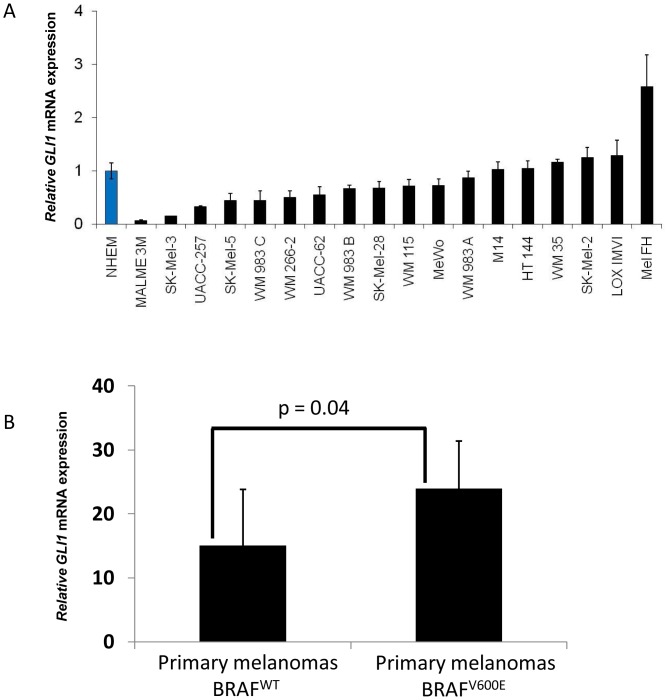
Evaluation of the *GLI1* expression in human melanoma cell lines and human primary melanoma tissues. **A**) Relative *GLI1* RNA expression in human melanoma cell lines and normal human melanocytes as measured by quantitative RT-PCR. **B**) *GLI1* RNA expression in human primary melanoma tissues harboring wild type (n = 7) or V600E activating mutation in BRAF (n = 15) evaluated by Affymetrix gene profiling. P<0.05 is considered significant. Mean values with SD are shown. ANOVA test with Tukey's post-test.

We performed quantitative RT-PCR to analyze the expression of the *GLI1* as a surrogate marker of Hedgehog signaling pathway activity in human melanoma cell lines. The expression of *GLI1* in majority of human melanoma cell lines including the ones from NCI-60 panel of human cancer cell lines (http://dtp.nci.nih.gov/docs/misc/common_files/cell_list.html) could be detected as in the case of the normal human melanocytes (Fig. 1 A).

Gene expression analysis was performed on our previously generated cDNA microarray (The data have been deposited in the National Center for Biotechnology Information GEO [51] and are accessible through GEO Series accession no. GSE8401), including primary melanoma tissues either being wild type for BRAF (n = 7) or harboring BRAF^V600E^ (n = 15) mutation using Affymetrix U133A Oligonucleotide Microarray platform. The expression of *GLI1* was significantly higher in tissue samples with BRAF^V600E^ mutation as compared to the wild type ones (Fig. 1 B).

### BRAF^V600E^ inhibitor PLX-4032 reduces GLI1 protein expression in human melanoma cells

**Figure 2:**
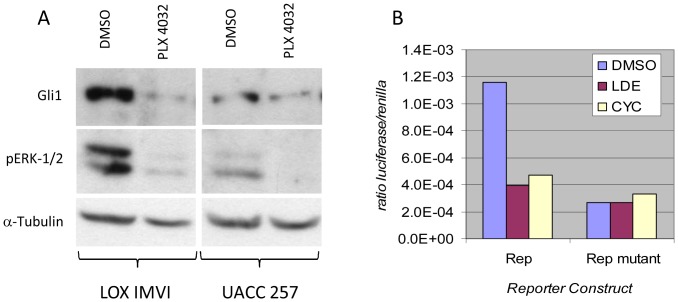
Inhibition of BRAF^V600E^, expression of GLI1 and SHH-GLI pathway inhibition by NVP-LDE225 in human melanoma cells *in vitro*. A) LOX IMVI and UACC 257 with PLX-4032 at the dose of 1 µM for 24 hr. subsequently protein lystaes prepared and subjected to WB analysis for the expression of GLI1 and phospho-ERK1/2. B) Effect of NVP-LDE225 on PTCH1 promoter. In total, 1 µg of *PTCH1* pGL3b-h*PTCH1*-prom-wt or pGL3b-h*PTCH1*-prom-mut luciferase construct and reporter were cotransfected into LOX IMVI cells. Cells were subsequently treated with 10 µM of NVP-LDE225 or cyclopamine for 4 hours (time point selected base on kinetic experiments). Fold activation was calculated relative to cells transfected with 3 µg of pB-actin-RL. One representative experiment of 2 is shown.

Human melanoma cell lines LOX IMVI and UACC 257 harboring BRAF^V600E^ mutation were treated with PLX-4032 and subsequently subjected to WB analysis for the expression of GLI1 and phospho-ERK 1/2 (as a marker for MAPK inhibition). The GLI1 expression was significantly reduced upon MAPK inhibition as was the expression of phospho-ERK 1/2 (Fig. 2A).

### NVP-LDE225 inhibits Hedgehog-GLI pathway in human melanoma cells *in vitro*

In order to study the effect of NVP-LDE225 [46] on the activity of the Hedgehog signaling pathway Luciferase Reporter Gene Assay was used. LOX IMVI human melanoma cells were transfected with either pGL3b-h*PTCH1*-prom-wt (Patched promoter containing 2 wild type GLI1 binding sites) or pGL3b-h*PTCH1*-prom-mut (Patched promoter containing 2 mutated GLI1 binding sites) and both containing the luciferase reporter sequence. In contrast to pGL3b-h*PTCH1*-prom-mut transfected, the pGL3b-h*PTCH1*-prom-wt LOX IMVI cells showed a significant decrease in luciferase activity after treatment with NVP-LDE225 or cyclopamine as compared to vehicle (DMSO) (Fig. 2B).

### NVP-LDE225 induces G1 cell cycle arrest in human melanoma cell lines *in vitro*

**Figure 3:**
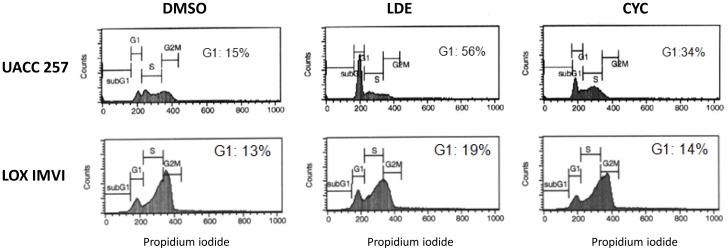
NVP-LDE225 and human melanoma cell cycle. Human melanoma cell lines were synchronized by thymidine and then treated with vehicle (DMSO), NVP-LDE225 or cyclopamine (each at 10 µM concentration). Cell cycle analysis performed 8 hr later. One representative experiment of 3 is shown.

Human melanoma cells were synchronized in G1-phase by using thymidine block. Cell cycle analysis performed 8 hr after treatment with NVP-LDE225, cyclopamine or DMSO (vehicle) showed significantly higher number of diploid 2 N cells (cells in G1 phase of the cell cycle) after NVP-LDE225 treatment as compared to vehicle (DMSO) (Fig. 3). The G1 cell cycle arrest after cyclopamine treatment was less pronounced than NVP-LDE225.

### NVP-LDE225 treatment results in decreased viability and induction of apoptotic cell death in human melanoma cell lines *in vitro*

**Figure 4:**
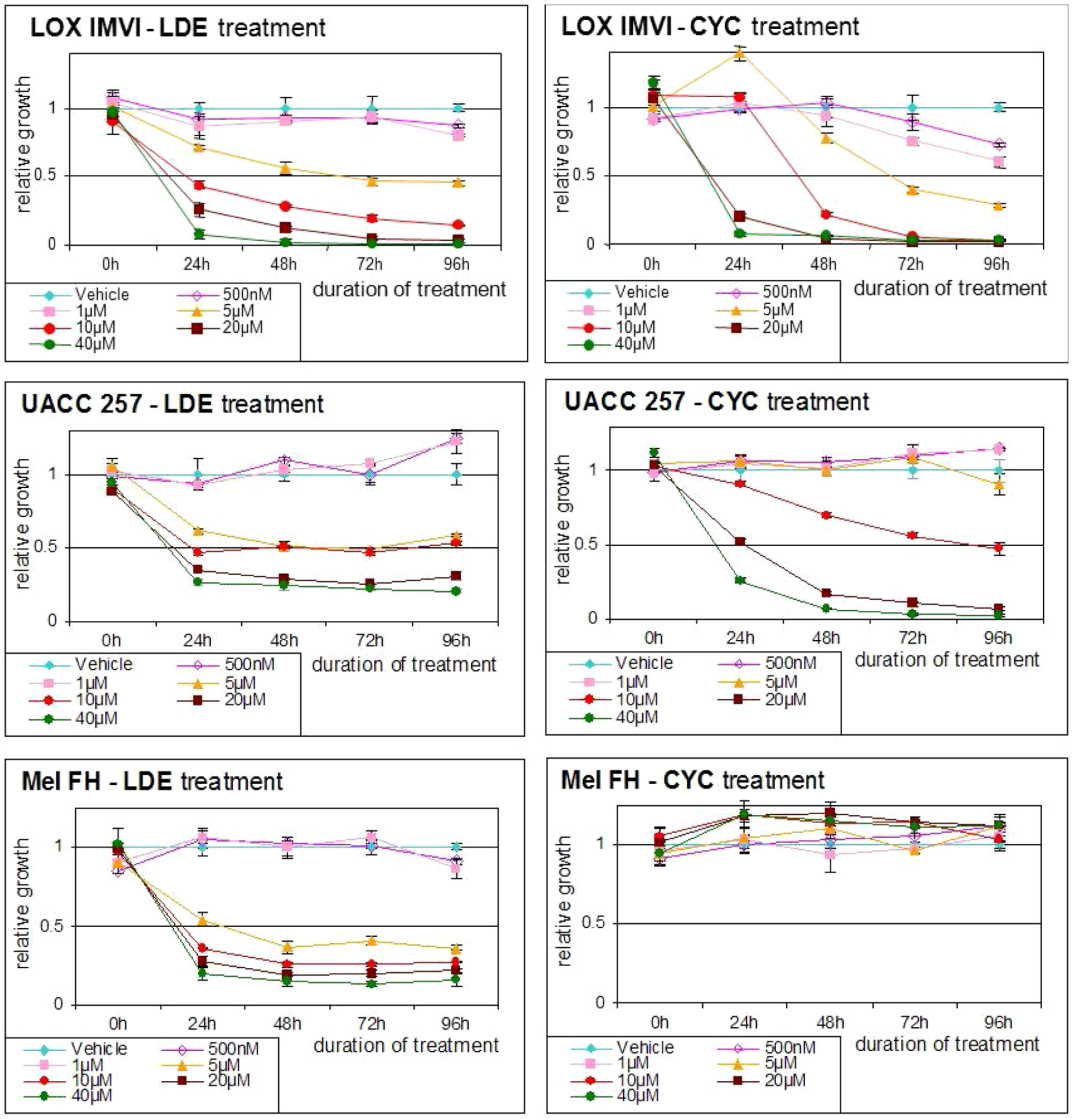
The effect of NVP-LDE225 on human melanoma cell viability. Human melanoma cell lines LOX IMVI, MEWO, SK-MEL-2, UACC 257, WM 115 and MEL FH were treated with different concentrations of NVP-LDE225, cyclopamine or the vehicle (DMSO). Cell viability at specific time points was measured by MTT assay. Experiments were performed in triplicates. One representative experiment is shown. Mean values with SD are shown.

Human melanoma cell lines were treated with different concentrations of NVP-LDE225, cyclopamine or the vehicle (DMSO). Viability of cells was assessed by MTT assay. There was a significant reduction of cell viability after treatment of cells with NVP-LDE225 and cyclopamine which was dose and time dependent. Interestingly, decreased tumor cell viability was more potent after NVP-LDE225 treatment as compared to after its natural counterpart cyclopamine treatment (Fig. 4 and Figure S2).

**Figure 5:**
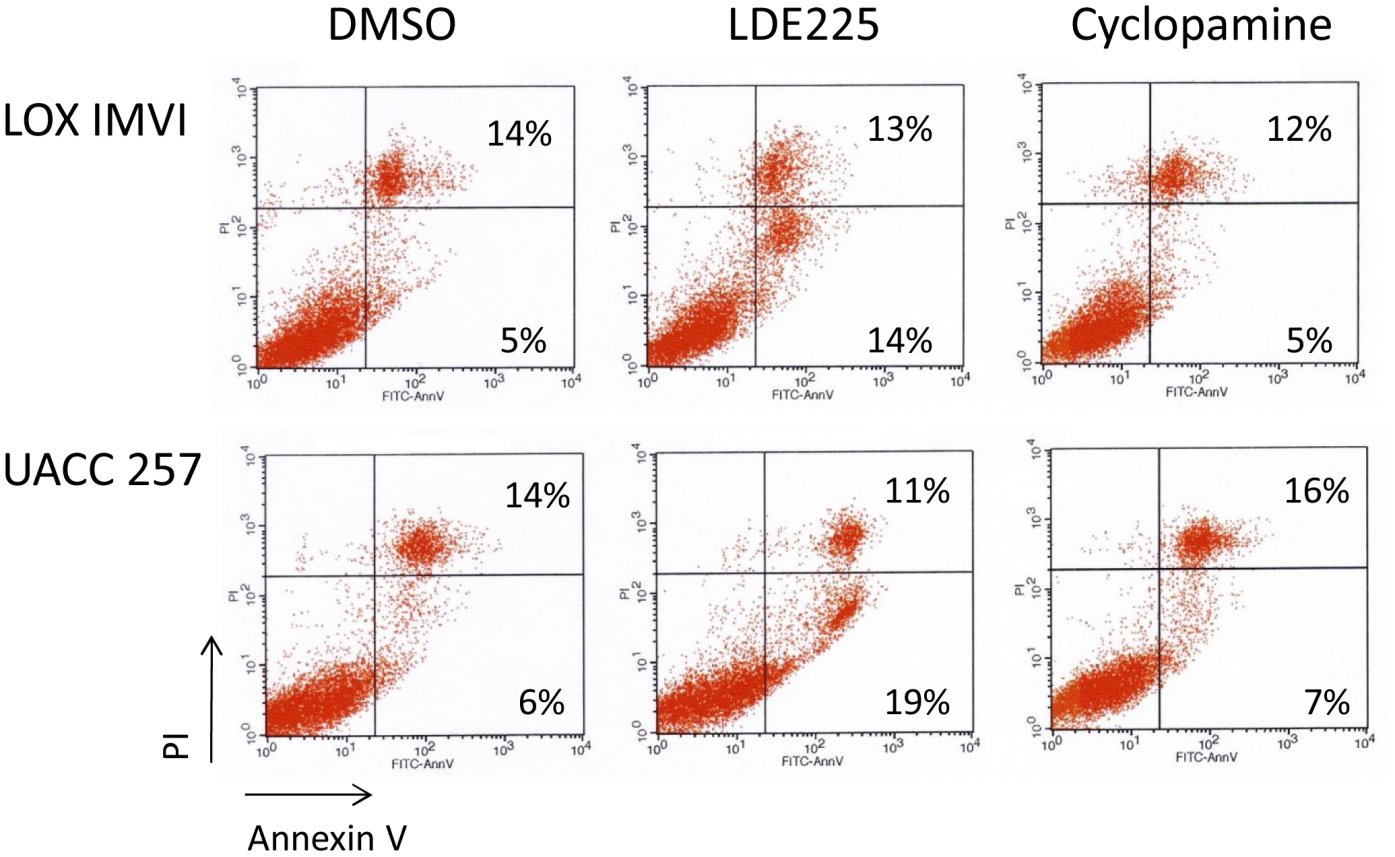
LDE225 induces apoptosis in human melanoma cell lines. Annexin V/PI staining of human melanoma cell lines after 48 hr of treatment with NVP-LDE225, cyclopamine (each at 10 µM concentration) or DMSO. Annexin V^+^/PI^−^ are apoptotic cells. Experiments were performed 3 times with similar results. One representative experiment is shown.

Decreased viability of human melanoma cell lines (LOX-IMVI, UACC-257 and MEL-FH) after NVP-LDE225 treatment was accompanied by changes in cell morphology including appearance of blebs and cell disintegration into apoptotic bodies (data not shown). This could be confirmed by annexin V staining where there was a significant induction of annexin V positive/propidium iodide negative apoptotic cells after NVP-LDE225, as compared to vehicle (DMSO) treatment (Fig. 5 and Figure S2). There was no induction of apoptosis after cyclopamine treatment at respected time point (48 hr). Dose response curves for cell growth to NVP-LDE225 or cyclopamine are demonstrated in Figure S3.

### NVP-LDE225 possesses antitumor activity against human melanoma and reduces GLI1 expression *in vivo*

**Figure 6:**
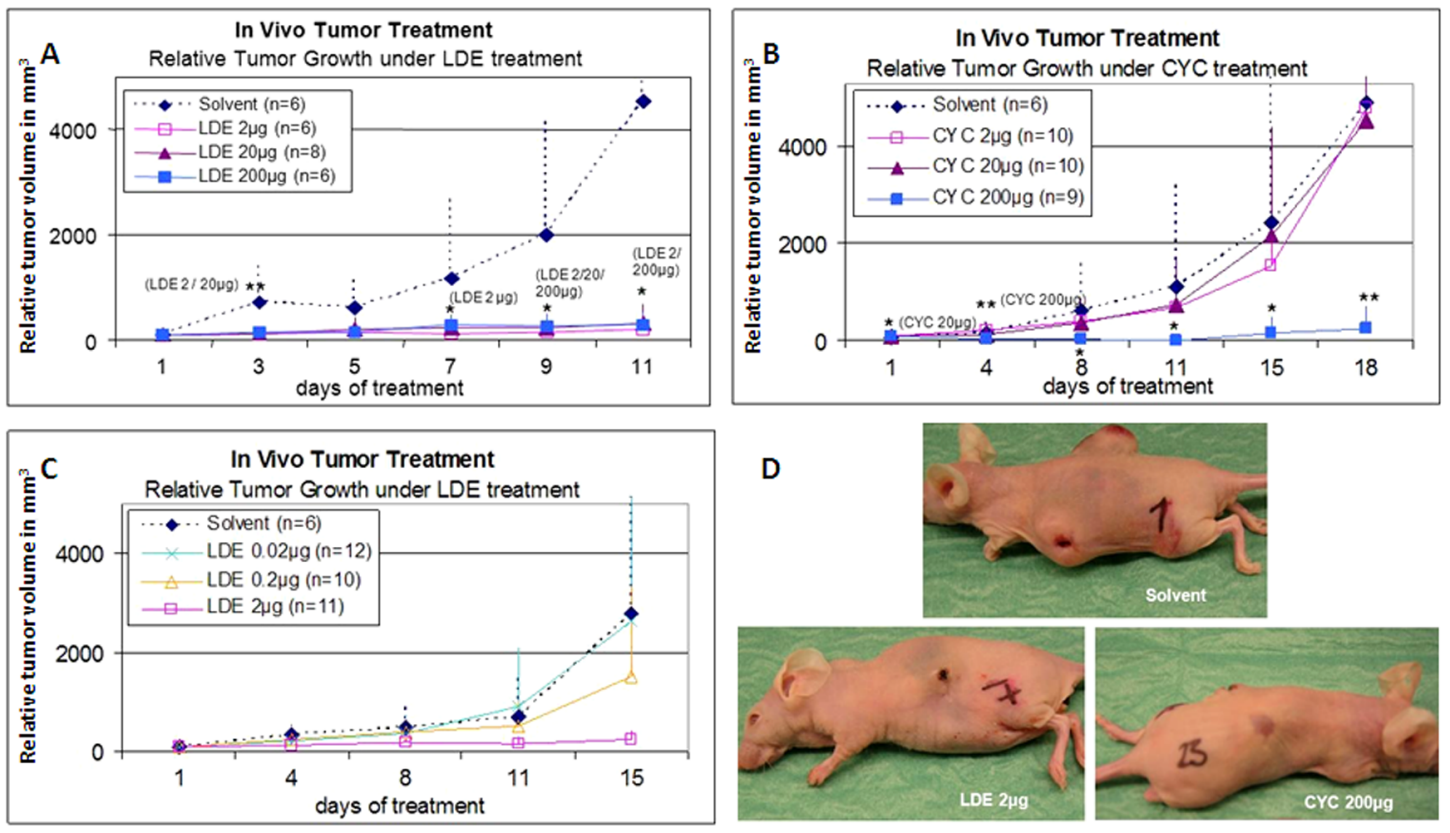
LDE225 antitumor activity in xenotransplantation model of human melanoma. **A & B**) 1×10^6^ LOX OMVI human melanoma cells suspended in PBS containing 10% FCS were injected s.c into both flanks. As tumors reach the mean volume of 48 mm^3^, NVP-LDE225 or cyclopamine were injected on daily basis at doses of 2, 20 or 200 µg/shot. (* p<0.05, ** p<0.01). **C**) 7.5×10^5^ of LOX IMVI cells were inoculated as above. Tumors with the mean volume of 18 mm^3^ were treated intratumorally on daily basis with NVP-LDE225 or vehicle. D) Photographs of mice after treatment with NVP-LDE225, cyclopamine or vehicle showing tumor volumes.

**Figure 7:**
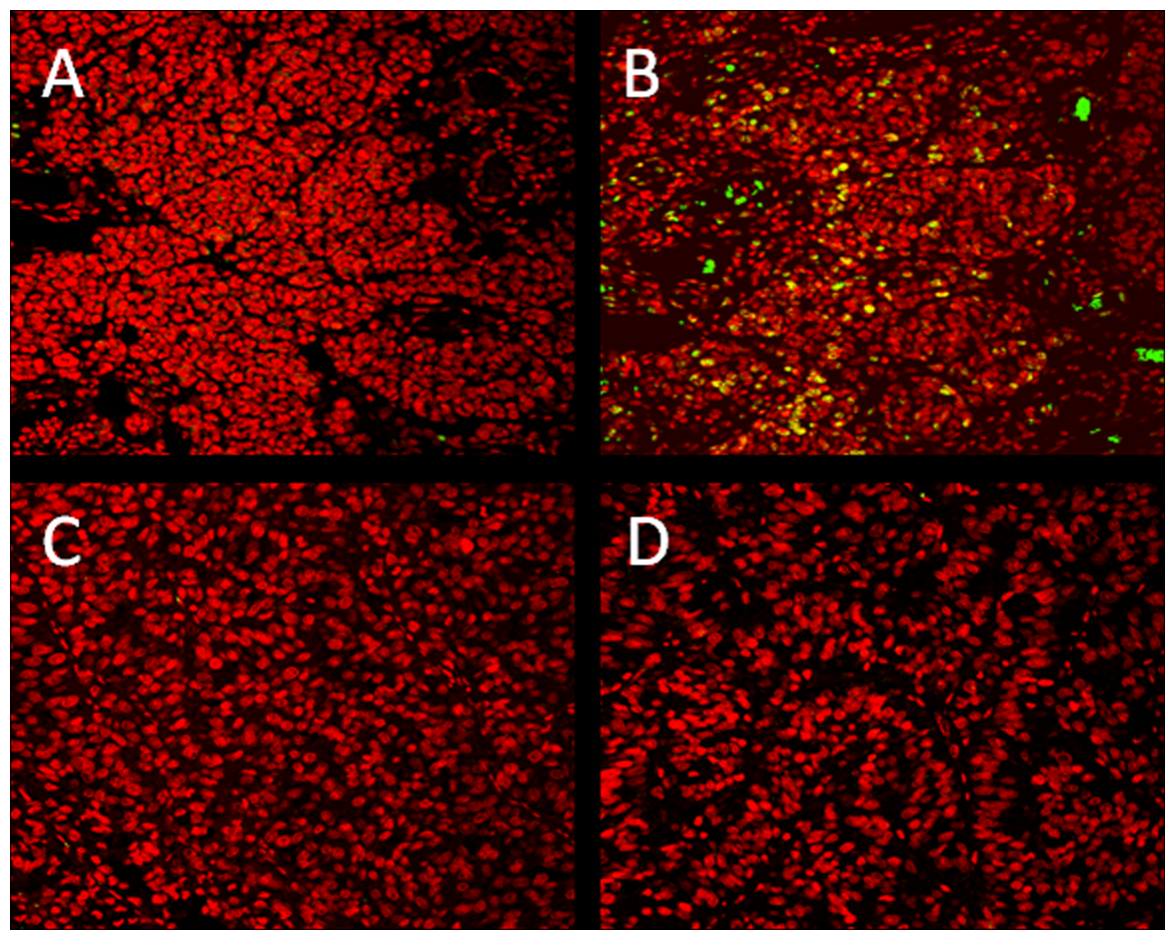
*In vivo* GLI1 expression after intratumoral administration of NVP-LDE225. LOX OMVI human melanoma cells were injected s.c into both flanks as mentioned above. Tumors were treated intratumorally on daily basis with vehicle (**A & B**) or NVP-LDE225 (**C & D**). Immunofluorescent microscopy of GLI1was performed on isolated tumor tissues. GLI1 staining was performed by overnight incubation of sections at 4°C with rabbit anti-human polyclonal Ab (**B & D**, NBP1-78259, Novus Biologicals, Littleton, CO) or isotype control (**A & C**) followed by an 1 hr-incubation with Alexa Fluor® 488 Donkey IgG, anti-rabbit (A21206, Invitrogen, Carlsbad, CA) at RT (green). Counterstaining of nuclei was performed with propidium iodide (red). Pictures were taken on a confocal laser-scanning microscope system (LSM 410; Zeiss). Yellow color corresponds to double positive (anti-GLI1 and propidium iodide) nuclear staining.

In the next step we investigated whether intratumorally administered NVP-LDE225 is able to inhibit the growth of human melanoma cells in athymic Nude-Foxn1 nu/nu mice. 1×10^6^ LOX IMVI human melanoma cells suspended in PBS containing 10% FCS were injected s.c into both flanks. As tumors reach the mean volume of 48 mm^3^, NVP-LDE225 or cyclopamine were injected on daily basis at doses of 2, 20 or 200 µg/shot. NVP-LDE225 as its natural counterpart cyclopamine induced a significant antitumor response (* p<0.05, ** p<0.01). Interestingly, 2 µg of NVP-LDE225 was as effective as 200 µg of cyclopamine (Fig. 6 A, B and D). Doses of NVP-LDE225 less than 2 µg, 0.2 and 0.02 µg showed non-significant or no antitumor response, respectively (Fig. 6 C). Both NVP-LDE225 and cyclopamine were well tolerated as mice showed no signs of weight loss or cachexia (data not shown). Immunofluorescent microscopy staining for GLI1 expression showed decreased expression upon NVP-LDE225 treatment but not vehicle (Fig. 7).

## Discussion

Multiple genetic alterations have been characterized in human melanoma [57]. Among them are the activating mutations in the NRAS (Q61R) and BRAF (V600E), the upstream MAPK signaling pathway members [57]. Recently, several studies have demonstrated that targeting BRAF^V600E^ in patients with this mutation is accompanied with a significant clinical response [9], [58], [59], [60]. As it has been the case for other oncogenic kinase inhibitors, response to PLX4032 is rapid but short lasting [60]. Some of the mechanisms that may underlie resistance to RAF inhibitors are de novo mutations in upstream kinase NRAS, spatial configuration of ARAF/BRAF/CRAF complex with subsequent activation of MEK-ERK pathway in a BRAF-independent manner, overexpression of mitogen-activated protein kinase kinase kinase 8 MAP3K8, or COT, which phosphorylates MEK in a RAF-independent manner and activation of other pathways (such as platelet derived growth factor receptor β (PDGFRβ, a receptor tyrosine kinase, is overexpressed in cellular models selected for RAF-inhibitor resistance in cell culture and in a subgroup of biopsy samples obtained from patients with progressing tumors) [61], [62], [63].

Based on these observations one can hypothesize that downstream targets of above mentioned signaling pathways could be more favorable for therapeutic applications which are largely unknown in human melanoma.

Recently, several reports have shown that the HH-GLI signaling pathway plays an important role in RAS-induced tumorigenesis (among them melanoma) and that MAPK signaling pathway regulates the expression of *GLI1* expression [37], [38], [40]. Stecca B. et al. [37] has reported that SHH-GLI signaling regulates the proliferation and survival of human melanoma *in vitro* and that growth, metastasis and recurrence in melanoma xenografts in mice are prevented by local or systemic interference of SHH-GLI function by using cyclopamine or siRNA against smoothened [37]. Moreover, others showed that oncogenic RAS-induced melanomas in transgenic mice express GLI1 and require SHH-GLI signaling *in vitro* and *in vivo* and that endogenous RAS-MEK and AKT signaling regulates the nuclear localization and transcriptional activity of *GLI1* in melanoma [37].

In our study the expression of the SHH-GLI signaling pathway target *GLI1* could be detected in 18 human melanoma cell lines and normal human melanocytes at mRNA level demonstrating the activity of this signaling pathway in human melanoma cell lines *in vitro*. This could be further confirmed *in vivo* in primary human melanomas and melanoma metastases by gene expression profiling (data not shown). Interestingly, in accordance with previous observations that MAPK signaling pathway leads to activation of HHG signaling pathway, the BRAF^V600E^ mutation in primary human melanomas was associated with significantly higher expression of *GLI1* as compared to the primary melanomas harboring wild type BRAF molecules. Consistently, inhibition of the BRAF^V600E^ in human melanoma cell lines using the specific inhibitor PLX-4032 (vemurafenib) resulted in substantial decrease in the expression of the downstream BRAF target phopho-ERK 1/2 as expected but also in GLI1 further confirming that GLI1 being a target of MAPK signaling pathway and that MAPK and SHH-GLI pathway do interact with each other in melanoma.

Cyclopamine (11-deoxojervine) is a natural chemical compound that belongs to the group of steroidal jerveratrum alkaloids. It is a teratogen isolated from the corn lily (veratrum californicum) that causes usually fatal birth defects. It can prevent the fetal brain from dividing into two lobes (holoprosencephaly) through inhibition of SHH-GLI and causes the development of a single eye (cyclopia). Cyclopamine is useful in studying the role of HH in the biology of cancer cells but due to its low specificity and side effects is not suitable for clinical use. Recently several novel and more specific small molecule inhibitors of SHH have been reported [64].

NVP-LDE225 has been shown to be a potent and specific inhibitor of smoothened [46]. It possesses antitumor activity *in vitro* and *in vivo* against a panel of human cancer cell lines [46]. Skvara et al. [65] has shown that this compound in a topical formulation has a significant antitumor activity against BCCs in Nevoid basal cell carcinoma syndrome (NBCCS) also known as Gorlin–Goltz syndrome [66]. A clinical trial testing the effect of oral formulation of this compound in the above mentioned group of patients is currently ongoing (personal communication).

In this study we show that NVP-LDE225 as well as the natural compound cyclopamine inhibit transcription of *GLI1* in human melanoma cell lines. NVP-LDE225 induces G1 cell cycle arrest and apoptosis. This is in line with the effects seen with MAPK inhibitors in melanoma cells [67] and cyclopamine in other cancer cell lines [68]. The GI_50_ dose of NVP-LDE225 in human melanoma cells was significantly lower than cyclopamine. Interestingly, the cell lines MEL FH and Mm329 being wild type for both BRAF and NRAS were also sensitive to NVP-LDE225 and the sensitivity was higher in comparison to cyclopamine. This needs further evaluation on more cell lines with similar geneotype and could potentially bear immense therapeutic benefit in BRAF^Wild Type^ patients which are completely resistant to vemurafenib. Finally, we demonstrate that NVP-LDE225 possesses a potent antitumor activity against human melanoma *in vivo*. This effect is significantly superior to the natural counterpart cyclopamine. The therapeutic effect of NVP-LDE225 was not associated with any visible adverse events in mice including weight loss or abnormal neurological behaviour.

Inhibition of SHH-GLI signalling pathway in BCC and medulloblastoma using specific inhibitors is accompanied by a significant clinical response [53], [65], [69], [70]. This has also resulted in the usage of these compounds against other solid and hematologic malignancies, either alone or in combination with other therapeutic strategies in the setting of clinical trials (http://clinicaltrials.gov/ct2/results?term=GDC-0449 and http://clinicaltrials.gov/ct2/results?term=LDE). In melanoma, a lack of complete clinical response (in patients harbouring BRAF^V600E^ mutation) to vemurafenib necessitates identification of novel therapeutic targets in melanoma. On the other hand there are no effective therapeutic strategies in patients with wild type BRAF tumors. Based on our data we can conclude that NVP-LDE2 can be potential therapeutic drug in human melanoma and is worth being further investigated.

## Supporting Information

Figure S1 **pGL3b-hPTCH1-prom-wt with Patched promoter containing 2 wild type GLI1 binding sites.** pGL3b-hPTCH1-prom-mut has two mutated Patched promoter GLI1 binding sites.
(DOCX)

Figure S2 **LDE225 induces apoptosis in human melanoma cell lines.** Annexin V/PI staining of human melanoma cell lines after 48 hr of treatment with NVP-LDE225, cyclopamine (each at 10 µM concentration) or DMSO. Annexin V+/PI− are apoptotic cells.
(DOCX)

Figure S3 **Dose response curves to NVP-LDE225 (LDE) or cyclopamine (CYC) are shown as percentage growth at 96 hr for cell lines used in this study.**
(DOCX)

Table S1 **Genetic characteristics of human melanoma cell lines used in this study.**
(DOCX)
