# Correction: NVP-LDE225, a Potent and Selective SMOOTHENED Antagonist Reduces Melanoma Growth *In Vitro* and *In Vivo*

**DOI:** 10.1371/annotation/ddd22094-5d8d-43ef-ad81-b95afe392ec7

**Published:** 2013-09-09

**Authors:** Ahmad Jalili, Kirsten D. Mertz, Julia Romanov, Christine Wagner, Frank Kalthoff, Anton Stuetz, Gaurav Pathria, Melanie Gschaider, Georg Stingl, Stephan N. Wagner

In the author byline the first two authors, Ahmad Jalili and Kirsten D. Mertz, should be noted as contributing equally.

Author Kirsten D. Mertz is not a corresponding author. The affiliations for this author were incorrect, and should be the following:

Division of Immunology, Allergy and Infectious Diseases, Department of Dermatology, Medical University of Vienna, Vienna, Austria

Current address: Cantonal Hospital Baselland, Institute for Pathology Liestal, Liestal, Switzerland 

