# Supplementary figures and images for: NVP-LDE225, a Potent and Selective SMOOTHENED Antagonist Reduces Melanoma Growth In Vitro and In Vivo
Source: PLoS One. 2013 Jul 30;8(7):e69064. doi: 10.1371/journal.pone.0069064 (PMC3728309; doi:10.1371/journal.pone.0069064)

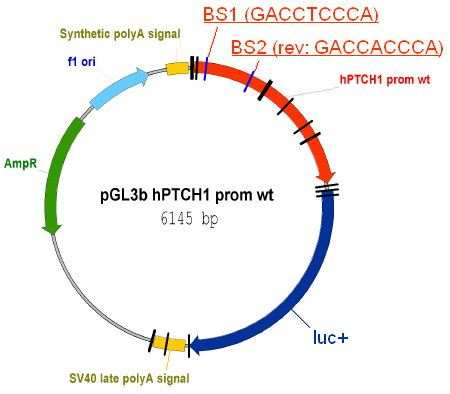

Supplement: Figure S1 — pGL3b-hPTCH1-prom-wt with Patched promoter containing 2 wild type GLI1 binding sites. pGL3b-hPTCH1-prom-mut has two mutated Patched promoter GLI1 binding sites. (DOCX) [file pone.0069064.s001.docx]

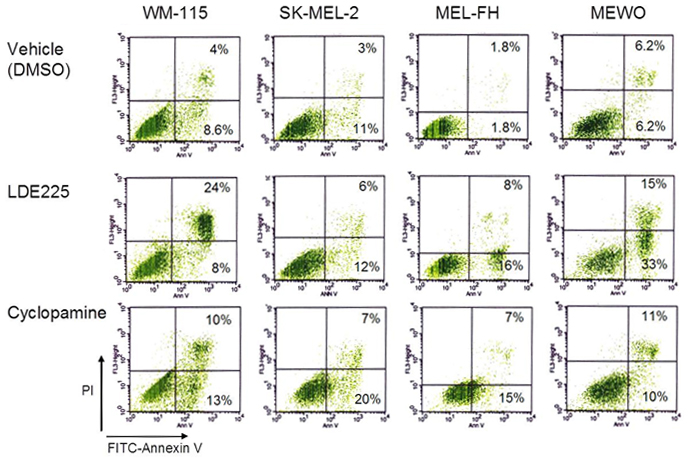

Supplement: Figure S2 — LDE225 induces apoptosis in human melanoma cell lines. Annexin V/PI staining of human melanoma cell lines after 48 hr of treatment with NVP-LDE225, cyclopamine (each at 10 µM concentration) or DMSO. Annexin V+/PI− are apoptotic cells. (DOCX) [file pone.0069064.s002.docx]

**
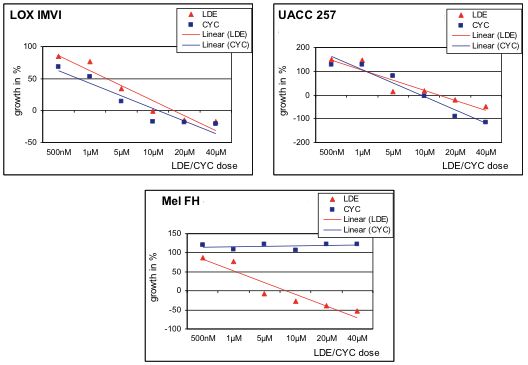
**

Supplement: Figure S3 — Dose response curves to NVP-LDE225 (LDE) or cyclopamine (CYC) are shown as percentage growth at 96 hr for cell lines used in this study. (DOCX) [file pone.0069064.s003.docx]

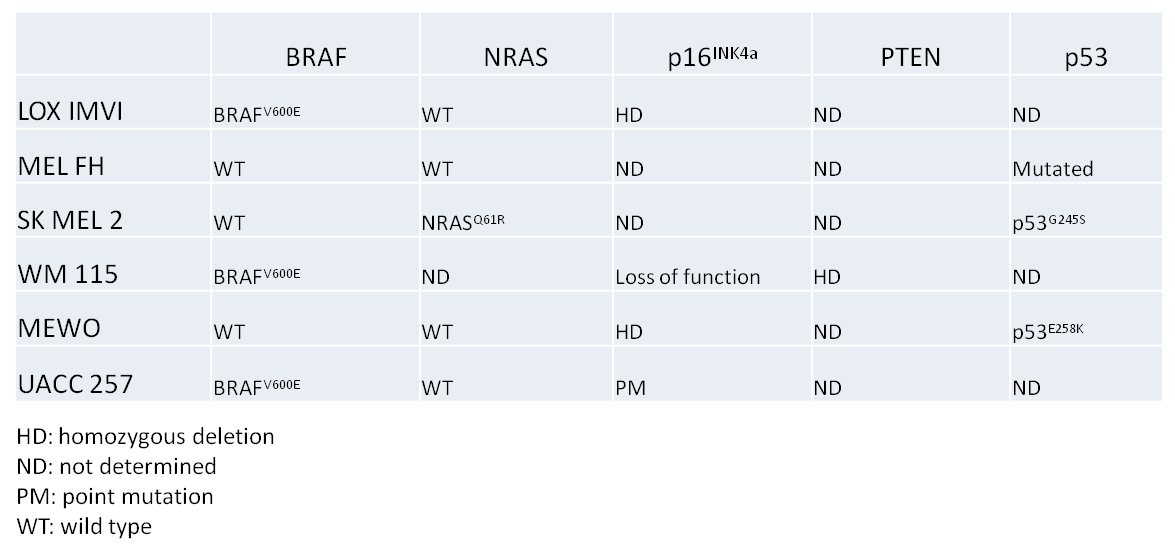

Supplement: Table S1 — Genetic characteristics of human melanoma cell lines used in this study. (DOCX) [file pone.0069064.s004.docx]
